# An extremely rare tetralogy of Fallot with absent pulmonary valve and unilateral absence of the pulmonary artery: a rare report of De Bucket Syndrome

**DOI:** 10.1186/s43044-025-00712-5

**Published:** 2026-01-09

**Authors:** Amirhossein Jalali, Mohammad Mahdavi, Mahmoud Ganjifard, Seyed Salaheddin Nabavi, Mohammad Bakhtiari, Zahra Ansari Aval, Seyyed Ebrahim Hosseini Zargaz

**Affiliations:** 1 Congenital Heart Diseases Research Center, Rajaie Cardiovascular Institute, Tehran, Iran; 2 Department of Anesthesiology and Critical Care, Faculty of Medicine, Birjand University of Medical Sciences, Birjand, Iran; 3 Department of General Surgery, School of Medicine,Imam Khomeini Hospital, Golestan Hospital, Ahvaz Jundishapur University of Medical Sciences, Ahvaz, Iran; 4 School of Medical Sciences, Sirjan School of Medical Sciences, Sirjan, Iran; 5 Cardiovascular Diseases Research Center, Birjand University of Medical Sciences, Birjand, Iran

**Keywords:** Tetralogy of Fallot, Absent pulmonary valve, Absent left pulmonary artery, Pulmonary regurgitation

## Abstract

**Background:**

Only a limited number of studies have reported on TOF with absent pulmonary valve (APV). Similarly, while cases of TOF with absent pulmonary artery (PA) have been documented, case reports describing TOF with both APV and absent PA are extremely rare.

**Case presentation:**

The present study investiged the case of a 1-year-old girl born at term with no initial clinical or physical signs of cyanosis. A subtle additional heart murmur detected during routine examination prompted referral to a cardiologist. Subsequent echocardiography and computed tomography (CT) angiography confirmed TOF with APV and absence of the left pulmonary artery (LPA). The patient later underwent corrective surgery, including pulmonary valve reconstruction and pulmonary artery plication.

**Conclusion:**

Although TOF is a common cyanotic congenital heart disease, certain variants of TOF, such as TOF with APV and absent LPA, may present without the typical cyanotic or respiratory symptoms. Therefore, even the slightest additional heart murmur should be thoroughly investigated. While clinical examination, arterial oxygenation, and echocardiography are essential, definitive diagnosis and precise anatomical characterization ultimately require CT angiography.

**Supplementary Information:**

The online version contains supplementary material available at 10.1186/s43044-025-00712-5.

## Introduction

Unilateral absence of a pulmonary artery (APA), also referred to as De Bücke’s syndrome, is a recognized form of congenital heart disease, occurring in approximately 0.6% of patients evaluated by cardiac catheterization [1]. Unilateral absence of the pulmonary artery (PA) occurs due to proximal sixth aortic arch coarctation and distal sixth aortic arch persistence as a patent ductus arteriosus. The incidence of absent pulmonary artery in tetralogy of Fallot (TOF) is 1%–3%, while absent pulmonary valve (APV) is reported to be 5%. However, the concurrent the Unilateral Absence of the Pulmonary Artery and pulmonary valve is extremely rare and complex, with approximately 50 cases reported worldwide so far [2, 3]. The landmark of this condition is aneurysmal dilatation of the main pulmonary artery and its branches, which can culminate in compression of the tracheobronchial tree and subsequent respiratory compromise [4]. The case we present is a 1-year-old infant who underwent successful surgery and was discharged after 10 days.

## Case report

A 12 month old female infant presented without overt respiratory distress or obvious cyanosis. Physical examination was the primary basis for clinical findings, and the patient’s oxygen saturation was 90%. The computed tomography (CT) angiography report indicated that the patient had a subaortic ventricular septal defect, APV, and absent left pulmonary artery (LPA). The right PA (RPA) ostium was stenotic, measuring 5.28 mm in diameter. Anthropometric data were as follows:

- Weight: 8 kg.
- Height: 72 cm.
- Body surface area (BSA): 0.44 m^2^.

**Fig. 1:**
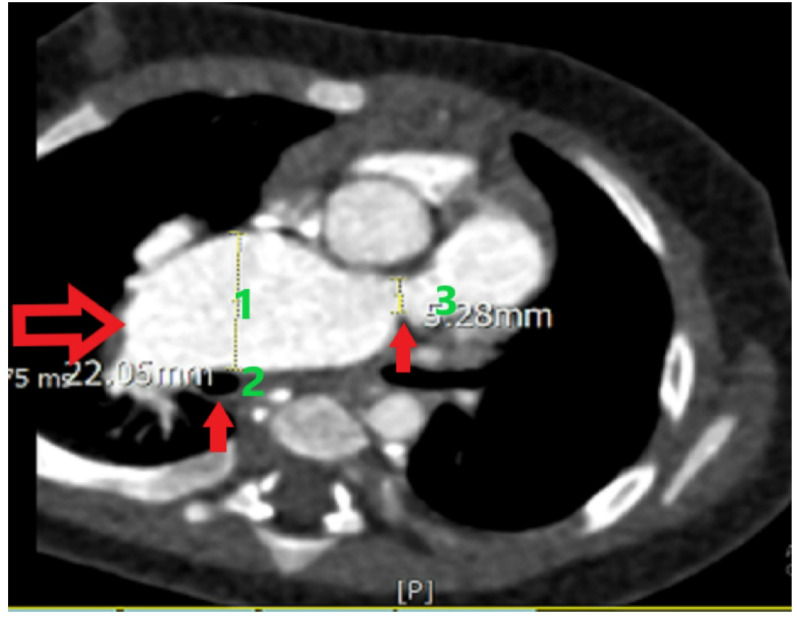
Anorismal RPA (1)-Right right bronchus (2) and pulmonary stenosis (3)

**Fig. 2:**
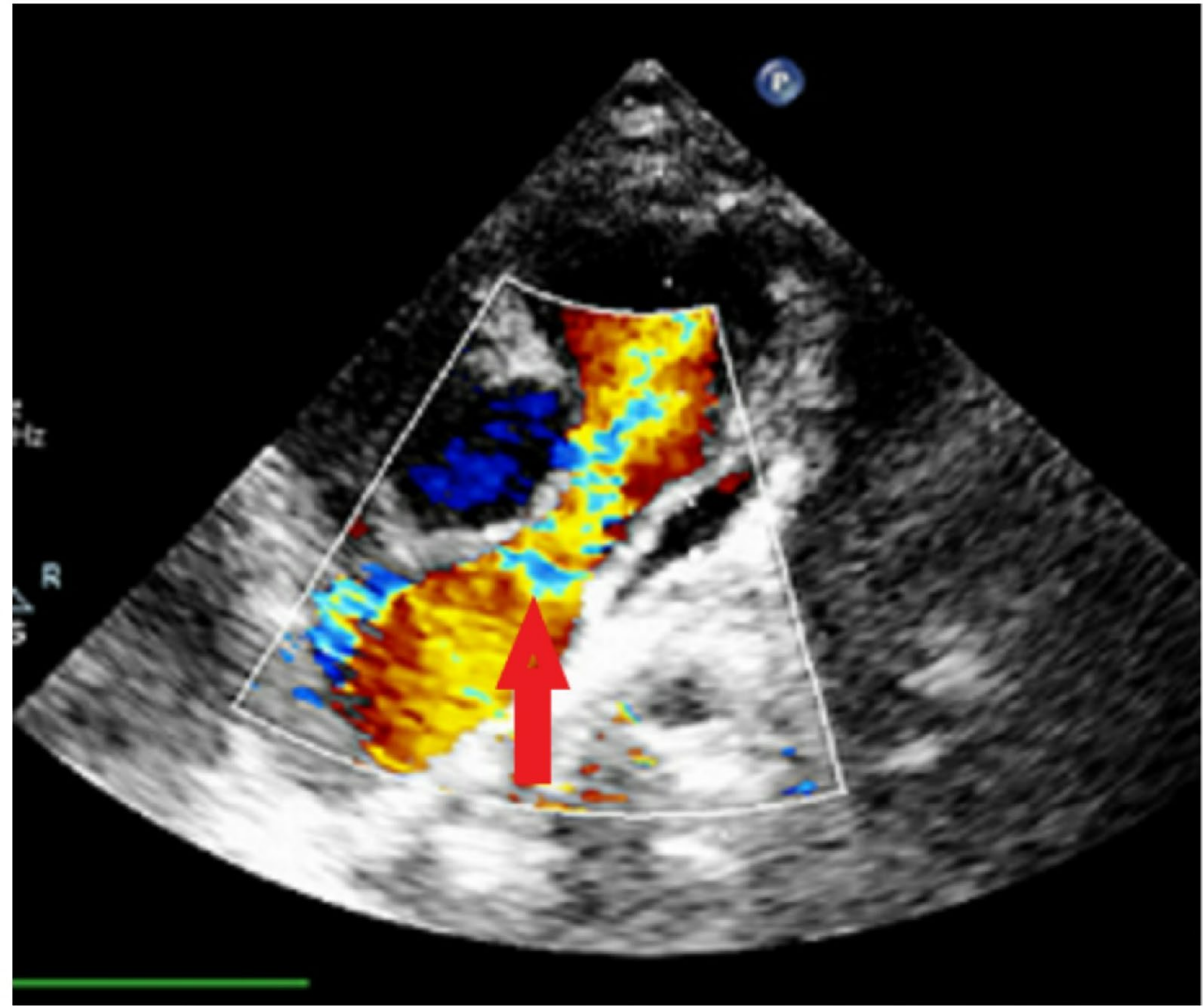
Currently of pulmonary stenosis and pulmonary insufficiency

Based on the BSA, the RPA ostial measurement corresponded to a Z-score of − 2.5 to − 3.0, while an aneurysmal mid-RPA diameter of 22.05 mm represented an estimated Z-score of + 4.0 to + 5.0. The mid and distal RPA showed aneurysmal dilation without thrombus. A PDA bump consistent with a closed ductal remnant was identified, and the aortic arch was left-sided (Figs. 1 and 2). While the patient did not exhibit severe respiratory symptoms, recovery from colds was prolonged, necessitating the use of respiratory inhalers. However, the CT did not reveal clear signs of airway compression. In such patients, we often observe varying degrees of bronchomalacia, meaning that even after relieving the dilatation and reducing airway compression, they may still experience some respiratory difficulties. To prevent future airway compression, anterior and posterior plication of the PA was entirely performed, along with the creation of a conduit between the RPA and the right ventricular outflow tract (RVOT) to prevent pulmonary valve insufficiency, because pulmonary regurgitation can exacerbate right ventricular failure and worsen PA dilatation; thus, both pulmonary valve and plication were taken into account as part of the surgical strategy. Ultimately, the patient underwent the RVOT shaving, closure of the patent foramen ovale (PFO), RPA plication, pulmonary valve replacement (14 mm conduit, Contegra/bovine jugular vein), and total thymectomy.

Given the presence of bronchomalacia, postoperative care included CPAP support in the ICU for a period after extubation to prevent bronchial collapse. Milrinone infusion was initiated before cardiopulmonary bypass to reduce pulmonary artery pressure, recognizing that all right ventricular output would perfuse a single lung. Separation from bypass was performed gradually, with measurement of right ventricular pressure at the end of surgery. In this case, right ventricular pressure was recorded at less than 70% of left ventricular pressure.

Postoperatively, the patient’s oxygen saturation improved from 90 to 100%, and she was discharged from the intensive care unit (ICU) after 1 week.

## Discussion

TOF is not only an anatomical abnormality but also appears to be a complex genetic disorder that can be accompanied by other anomalies, such as APV, absent PA, and extracardiac abnormalities [1]. Symptoms of TOF with APV were first described by Chevers in 1847. Due to APV, a high-flow blood stream enters the main PA and its branches. This increased diameter puts pressure on the bronchi and consequently leads to respiratory impairment [3, 5–7]. Currently, as with most congenital heart diseases, there is no good palliative treatment for TOF with APV, and the surgery timing depends on the patient’s symptoms. Patients with APV present with either respiratory symptoms or no respiratory symptoms. The results of a study involving 6 surgeries on patients undergoing TOF with APV revealed that all patients had respiratory symptoms. However, in our case, the patient did not exhibit respiratory symptoms [3]. Several studies have shown that preoperative ventilation and surgery in infants significantly impact postoperative ventilation time and survival rates. In our case, due to the lack of significant respiratory symptoms, mechanical ventilation was not performed. The mortality rate in infancy is over 50%, while the mortality rate in infants and children declines to less than 20%. Hence, surgery in infancy is not recommended due to the high risk of adverse outcomes [3]. For surgical methods, pulmonary arterioplasty, pulmonary valvuloplasty, or complete replacement of the central PA with valved bovine jugular vein can be performed to treat dilated PA and APV. Although pulmonary valve insufficiency can be more effectively corrected through PA replacement compared to pulmonary arterioplasty and valvuloplasty, PA replacement may increase operative time and the need for repeated interventions. Other surgeries can be performed concurrently to repair structural heart abnormalities, such as TOF [1, 3].

In our case, the pulmonary valve was made, and due to the narrow PA collaterals, RPA plication was performed. Unilateral absence of the LPA is five to eight times more common than the RPA. Some patients, despite the apparent absent PA, may have a latent PA (normal or small in size) with a tight connection between the ductus arteriosus and the PA. In our case, the LPA was also absent, and there was no connection between the ductus arteriosus and the PA. However, due to the formation of collaterals, there was a minimal blood supply to the left lung. Given their very small diameter (1 mm), it was not possible to perform unifocalization [8, 9]. Symptoms of TOF with APV typically include mild cyanosis and mostly respiratory distress. The respiratory distress is attributed to the high pressure of the LPA on the left main bronchus. Since the LPA was absent in our case, our patient did not experience respiratory symptoms [3].

## Conclusion

Despite the absence of clinical cyanotic symptoms and respiratory difficulties in our case, the presence of an additional heart murmur prompted further investigation with echocardiography and CT angiography. These investigations led to the diagnosis of TOF with APV and absent LPA. It is crucial to note that in cyanotic heart conditions, such as TOF, reliance solely on cyanotic symptoms in clinical examinations is insufficient because some cases of TOF, like our case with absent pulmonary valve, may exhibit very mild cyanotic symptoms due to severe pulmonary valve regurgitation rather than severe pulmonary stenosis. Hence, even the slightest additional heart murmur should prompt more precise investigations using echocardiography and CT angiography to thoroughly assess intracardiac and extracardiac shunts and congenital anomalies. Evidence from recent series and our current case support that tailored surgical correction can restore pulmonary perfusion, improve oxygenation, and prevent long term complications, thereby offering a definitive and durable treatment option for this rare anomaly.

## Supplementary Information

Below is the link to the electronic supplementary material.

Supplementary Material 1.

## Data Availability

No datasets were generated or analysed during the current study.

## Abbreviations

APV: Absent pulmonary valve
TF: Tetralogy of Fallot
RVOT: Right ventricular outflow tract
