# Supplementary material for: An extremely rare tetralogy of Fallot with absent pulmonary valve and unilateral absence of the pulmonary artery: a rare report of De Bucket Syndrome
Source: Egypt Heart J. 2026 Jan 9;78:2. doi: 10.1186/s43044-025-00712-5 (PMC12789335; doi:10.1186/s43044-025-00712-5)
Supplement: Supplementary file 1 — Supplementary Material 1. [file 43044_2025_712_MOESM1_ESM.pdf]

CARE case report guidelines

!!!CARE!Checklist!of!information!to!include!when!writing!a!case!report!

(cc) BY-NC-ND

✓

Topic	Item	Checklist item description	Reported on Line
**Title**	1	The diagnosis or intervention of primary focus followed by the words "case report"	2-3
**Key Words**	2	2to 5 key words that identify diagnoses or interventions in this case report, including "case report"	19-20
**Abstract (no references)**	3a	Introduction: What is unique about this case and what does it add to the scientific literature?	~~6-8~~
	3b	Main symptoms and/or important clinical findings	9-10
	3c	The main diagnoses, therapeutic interventions, and outcomes	10-13
	3d	Conclusion—What is the main "take-away" lesson(s) from this case?	~~15-18~~
**Introduction**	4	One or two paragraphs summarizing why this case is unique (**may include** references)	22-30
**Patient Information**	5a	De-identified patient specific information.	31
	5b	Primary concerns and symptoms of the patient.	32
	5c	Medical, family, and psycho-social history including relevant genetic information	33
	5d	Relevant past interventions with outcomes	34
**Clinical Findings**	6	Describe significant physical examination (PE) and important clinical findings.	35
**Timeline**	7	Historical and current information from this episode of care organized as a timeline	36
**Diagnostic Assessment**	8a	Diagnostic testing (such as PE, laboratory testing, imaging, surveys).	37
	8b	Diagnostic challenges (such as access to testing, financial, or cultural)	38
	8c	Diagnosis (including other diagnoses considered)	39
	8d	Prognosis (such as staging in oncology) where applicable	40
**Therapeutic Intervention**	9a	Types of therapeutic intervention (such as pharmacologic, surgical, preventive, self-care)	41
	9b	Administration of therapeutic intervention (such as dosage, strength, duration)	~~42~~
	9c	Changes in therapeutic intervention (with rationale)	~~43~~
**Follow-up and Outcomes**	10a	Clinician and patient-assessed outcomes (if available)	~~44~~
	10b	Important follow-up diagnostic and other test results	~~45~~
	10c	Intervention adherence and tolerability (How was this assessed?)	~~46~~
	10d	Adverse and unanticipated events	47
**Discussion**	11a	A scientific discussion of the strengths AND limitations associated with this case report	48
	11b	Discussion of the relevant medical literature **with references**.	50
	11c	The scientific rationale for any conclusions (including assessment of possible causes)	51
	11d	The primary "take-away" lessons of this case report (without references) in a one paragraph conclusion	85-95
**Patient Perspective**	12	The patient should share their perspective in one to two paragraphs on the treatment(s) they received	
**Informed Consent**	13	Did the patient give informed consent? Please provide if requested	Yes ■ No ■
